# Differential effects of nAChR modulators on impulsive action in a nicotine self-administration Go/No-Go task in rats

**DOI:** 10.3389/fphar.2026.1883945

**Published:** 2026-09-21

**Authors:** Ranjithkumar Chellian, Guido Huisman, Lara Caglayan, Adriaan W. Bruijnzeel

**Affiliations:** 1 Department of Psychiatry, University of Florida, Gainesville, FL, United States; 2 Department of Biomedical Sciences, Texas A&M University, Dallas, TX, United States

**Keywords:** Go/No-Go procedure, Go/No-Go task, impulsive action, impulsive actions, impulsivity, inhibitory control, mecamylamine, nAChRs

## Abstract

**Introduction:**

Tobacco use disorder is a chronic condition characterized by compulsive nicotine use, withdrawal, and relapse following abstinence. Impulsivity contributes to persistent nicotine use and poor cessation outcomes. This study examined whether nicotinic acetylcholine receptor (nAChR) modulators alter impulsive action in a nicotine self-administration Go/No-Go task in male and female rats.

**Methods:**

Rats acquired intravenous nicotine self-administration and were then trained in a Go/No-Go procedure in which active lever presses were reinforced during Go periods but not during No-Go periods. Impulsive action was quantified as the percentage of active lever responses during No-Go periods relative to total active responses. We then assessed the effects of varenicline (0.1–3 mg/kg), nicotine (0.1–0.6 mg/kg), and the nAChR antagonist mecamylamine (0.5–2 mg/kg) in the Go/No-Go procedure.

**Results:**

Varenicline and nicotine pretreatment reduced active responding during both Go and No-Go periods, whereas mecamylamine selectively reduced responding during No-Go periods. Mecamylamine decreased the percentage of active responses during No-Go trials, indicating reduced impulsive action during nicotine self-administration. In contrast, nicotine and varenicline did not alter response allocation, suggesting that their effects reflected nonspecific reductions in responding rather than changes in impulsive action. No sex differences were observed. Substituting saline for nicotine during self-administration did not alter active responding during Go periods, but rats in the saline group had fewer active responses during No-Go periods than rats in the nicotine group.

**Discussion:**

These results demonstrate differential effects of nAChR modulators on impulsive action during nicotine self-administration. This work supports the utility of Go/No-Go self-administration task for investigating nAChR-dependent modulation of impulsive action during nicotine self-administration.

## 1 Introduction

Tobacco use disorder is a chronic condition characterized by the persistent, problematic use of tobacco products that disrupts daily functioning and leads to emotional distress (American Psychiatric Association, 2013). Specifically, smokers may experience a loss of control over tobacco use, a persistent desire or unsuccessful efforts to quit, craving, continued use despite adverse consequences, and withdrawal (American Psychiatric Association, 2013). Worldwide, approximately 1.25 billion people smoke, and tobacco use remains the leading preventable cause of morbidity and mortality (World Health Organization, 2024). Smoking is associated with a broad spectrum of diseases, including cardiovascular disease, respiratory disease, cancer, and Alzheimer’s disease (Ott et al., 1998; Ambrose and Barua, 2004; Sasco et al., 2004; Forey et al., 2011). In addition to its adverse health consequences, tobacco use is a significant economic burden due to an increase in medical expenses and lost productivity (Goodchild et al., 2018; Nargis et al., 2022). Despite the availability of FDA-approved smoking cessation aids and widespread public health initiatives, smoking and relapse rates remain high (Williams et al., 2007; Agboola et al., 2015; Cornelius et al., 2023).

Impulsivity plays a critical role in the initiation, maintenance, and relapse of tobacco use (Doran et al., 2004; Perry and Carroll, 2008). Impulsivity is a tendency toward rapid, often poorly considered actions that can have negative consequences for oneself or others (Moeller et al., 2001). Impulsivity encompasses several distinct components, including response inhibition (inability to withhold a response), delay discounting (preferring immediate, smaller rewards over delayed, larger rewards), and attentional impulsivity (tendency to act without focus) (Weafer et al., 2011; Jauregi et al., 2018). A deficit in the inhibition of responses has been associated with substance abuse, including tobacco use disorder (Smith et al., 2014). This study focuses specifically on impulsive action, which is operationally defined as a failure of response inhibition or the inability to withhold a prepotent motor response. The Go/No-Go task is a classic paradigm for assessing this behavior, requiring subjects to inhibit a response in the presence of a ‘No-Go’ signal despite the prepotent drive to respond established during alternating ‘Go’ periods. The Go/No-Go task has been widely used in humans and animal studies to assess inhibitory control (Casey et al., 1997; Rubia et al., 2001; Durston et al., 2002; Gubner et al., 2010), specifically by measuring the inhibition of motor responses before they begin (Eagle et al., 2008). Tobacco users have diminished inhibitory control in the Go/No-Go task compared to nonsmokers (Luijten et al., 2011). ​In Go/No-Go procedures, responding during No-Go periods reflects failures of response inhibition (impulsive action). Additionally, the percentage of active responses during No-Go periods can be used as a normalized measure of failures to withhold responding in Go/No-Go procedures (Kolokotroni et al., 2011; Zapata et al., 2017; Zapata and Lupica, 2021). Pharmacological treatments, neuromodulation techniques, and behavioral therapies that improve impulse control have shown efficacy in reducing substance use and relapse among individuals with substance use disorders (Goldstein and Volkow, 2011; Zilverstand et al., 2018; Steele et al., 2019). For instance, transcranial direct current stimulation (tDCS) has shown promise in enhancing inhibitory control and decreasing relapse rates in people with alcoholism by modulating activity in the dorsolateral prefrontal cortex (Dubuson et al., 2021).

Nicotine is a nonselective agonist at nicotinic acetylcholine receptors (nAChRs) and mediates its rewarding effects by activating α4β2, α3β4, α7, and α6-containing nAChRs (Picciotto and Kenny, 2021). Animal models play a critical role in improving our understanding of the neurobiological mechanisms underlying nicotine self-administration, withdrawal, and relapse (Jin et al., 2020; Chellian et al., 2024b). These models recognize that drug-seeking is driven by both the pharmacological effects of nicotine and the powerful influence of drug-associated cues (Donny et al., 2003; Caggiula et al., 2008). Rats readily learn to self-administer nicotine, and nicotine has rewarding properties in well-established assays such as conditioned place preference and intracranial self-stimulation (Corrigall and Coen, 1989; Le Foll and Goldberg, 2005; Chellian et al., 2021; Chellian et al., 2025). Furthermore, cessation of nicotine intake leads to withdrawal and animals display nicotine seeking behavior after a period of abstinence (Chellian et al., 2024b). Sex differences in nicotine self-administration and relapse-related behavior have also been reported in rats, with females showing greater nicotine intake under extended-access conditions and greater nicotine seeking after forced abstinence (Chellian et al., 2024b). Animal models have also been developed to study impulsivity and response inhibition and allow for the investigation of the neurobiological mechanisms that mediate increased impulsivity in smokers (Eagle et al., 2008; Winstanley, 2011). The rodent Go/No-Go task requires animals to discriminate between cues signaling reward delivery (Go) and cues indicating reward omission (No-Go) and thereby enables the assessment of inhibitory control. Kolokotroni et al. assessed impulsivity using a symmetrically reinforced Go/No-Go task, in which both correct responses during Go trials and successful inhibition during No-Go trials were rewarded with sucrose pellets (Kolokotroni et al., 2011). They found that acute noncontingent nicotine administration impaired response inhibition in rats (Kolokotroni et al., 2011).

Prior studies have established that acute, non-contingent nicotine administration impairs response inhibition in tasks such as the Go/No-Go and the 5-Choice Serial Reaction Time Task (5-CSRTT) (Blondel et al., 2000; Kolokotroni et al., 2011). However, the influence of nicotine on impulsive action within a voluntary self-administration context, where drug-taking is actively maintained, remains less clear. The primary advantage of integrating a Go/No-Go task with drug self-administration is that it allows for the study of impulsive action in animals motivated to take the drug, which more closely models the human condition of tobacco use disorder.

Signaled drug-unavailable periods were initially used by Deroche-Gamonet et al. to assess persistence in drug seeking, and were subsequently adapted into a Go/No-Go cocaine self-administration procedure by Zapata et al. (Deroche-Gamonet et al., 2004; Zapata et al., 2017). We adapted this procedure to examine impulsive action during nicotine self-administration (Zapata et al., 2017; Zapata and Lupica, 2021). In this framework, responding during Go periods reflects nicotine-maintained responding, whereas responding during No-Go periods reflects failure to withhold a previously reinforced drug-directed response when nicotine is unavailable. We therefore use No-Go responding as a measure of impulsive action expressed in the context of drug seeking. This design enabled the simultaneous assessment of nicotine-maintained responding during Go periods and response inhibition during No-Go periods within the same session. Rats were trained to self-administer nicotine, and we assessed nicotine intake during the Go periods and lever responding during the No-Go periods as a measure of impulsive action. To investigate the role of FDA-approved smoking cessation treatments and nAChRs in impulsive behavior, we examined the effects of pretreatment with varenicline (Chantix), mecamylamine, and nicotine on performance in the Go/No-Go task. Varenicline is a partial agonist at α4β2 nAChRs and a full agonist at α7 receptors (Mihalak et al., 2006). Varenicline is an FDA-approved smoking cessation treatment that reduces nicotine craving and withdrawal (Gonzales et al., 2006). A study with smokers suggests that varenicline may improve attention without affecting impulsive action (Lesage et al., 2020). Mecamylamine is a non-selective nAChR antagonist that has been shown to attenuate the behavioral and reinforcing effects of nicotine (Lundahl et al., 2000; Nickell et al., 2013). Several studies have indicated that mecamylamine may aid in smoking cessation, particularly when combined with nicotine replacement therapy (NRT) (Rose et al., 1994; Lancaster and Stead, 1998). Nicotine replacement is another FDA-approved treatment for smoking cessation (Stead et al., 2012). Therefore, we also investigated the effects of nicotine pretreatment on responding for nicotine and impulsive action during the Go/No-Go test. By assessing the effects of these compounds on performance in the Go/No-Go task, we aimed to determine how modulation of nAChR signaling affects impulsive action after nicotine self-administration.

## 2 Materials and methods

### 2.1 Animals

Adult male (220–280 g, 8–9 weeks of age; N = 12) and female (180–225 g, 8–9 weeks of age; N = 12) Wistar rats were purchased from Charles River (Raleigh, NC). The rats were housed with a rat of the same sex in a climate-controlled vivarium on a reversed 12 h light-dark cycle (light off at 7 a.m.). The rats were handled for 2–3 min per day for several days before surgery. Following jugular catheter implantation, rats were singly housed for the remainder of the study. Food and water were available *ad libitum* in the home cage throughout the study. The experimental protocols were approved by the University of Florida Institutional Animal Care and Use Committee (IACUC). All experiments were performed in accordance with relevant IACUC guidelines and regulations and in compliance with ARRIVE guidelines 2.0 (Animal Research: Reporting of *In Vivo* Experiments).

### 2.2 Drugs

For intravenous self-administration (−)-nicotine hydrogen tartrate (NIDA Drug Supply Program) was dissolved in sterile saline (0.9% sodium chloride), and the pH was adjusted to 7.2 ± 0.2 using 1 M NaOH. Rats self-administered nicotine at a dose of 0.03 mg/kg/inf (expressed as base) in a volume of 0.1 mL/inf. For drug treatments (−)-nicotine hydrogen tartrate, varenicline tartrate (Tocris bioscience, Minneapolis, MN), and mecamylamine hydrochloride (NIDA Drug Supply Program) were dissolved in sterile saline and administered subcutaneously (SC) in a volume of 1 mL/kg body weight. Nicotine doses are expressed as base, while varenicline and mecamylamine doses are expressed as salt.

### 2.3 Experimental design

A schematic overview of the experimental timeline is presented in Figure 1. Rats were surgically implanted with catheters in the jugular vein and allowed a minimum 7-day recovery period. Following recovery, male (N = 12) and female (N = 12) rats were trained to self-administer nicotine during fifteen 2 h sessions. This was followed by 41 days of nicotine self-administration under a Go/No-Go training paradigm. After completion of the Go/No-Go training sessions, the effects of varenicline, nicotine, and mecamylamine on nicotine self-administration in the Go/No-Go task were evaluated. In addition, a separate comparison examined responding in rats self-administering saline under the Go/No-Go schedule.

**FIGURE 1:**
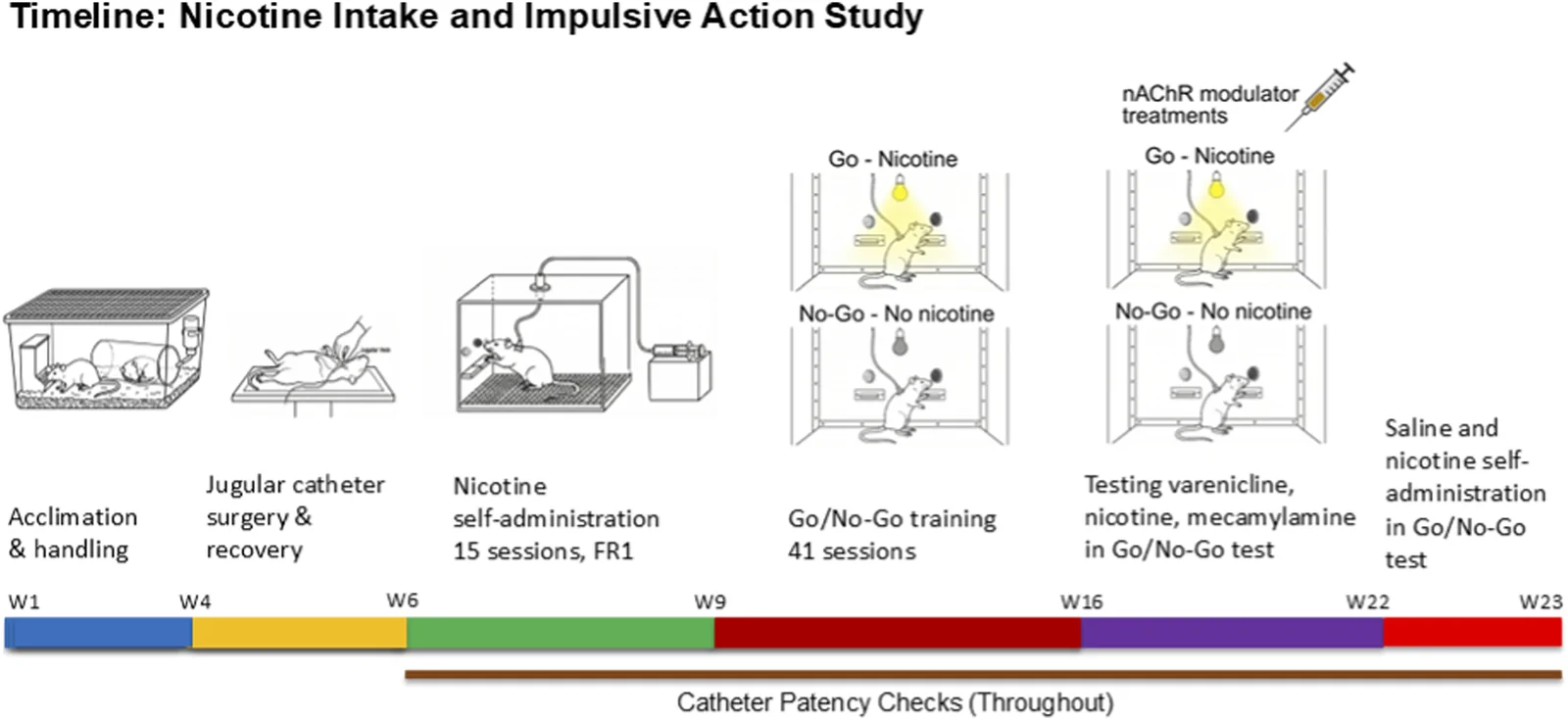
Schematic overview of the experimental timeline for the Go/No-Go nicotine self-administration study. Male and female rats were acclimated and handled prior to implantation of the jugular catheter followed by a minimum 7-day recovery period. Rats then acquired intravenous nicotine self-administration during fifteen 2-h sessions under an FR1 schedule. This was followed by 41 sessions of nicotine self-administration under a Go/No-Go training paradigm with alternating Go and No-Go periods. After completion of the Go/No-Go training, the effects of varenicline, nicotine, and mecamylamine on performance in the Go/No-Go task were assessed. In a separate comparison, rats self-administered saline under the Go/No-Go schedule to allow comparison with nicotine self-administration. Catheter patency was monitored throughout the study. Males, N = 12; Females, N = 12.

### 2.4 Intravenous catheter implantation

The catheters were implanted as described before (Chellian et al., 2024a; Chellian et al., 2024b). The rats were anesthetized with an isoflurane-oxygen vapor mixture (1%–3%) and prepared with a catheter in the right jugular vein. The catheters consisted of polyurethane tubing (length 10 cm, inner diameter 0.64 mm, outer diameter 1.0 mm, model 3Fr, Instech Laboratories, Plymouth Meeting, PA). The right jugular vein was isolated, and the catheter was inserted 2.9 cm for males and 2.5 cm for females. The tubing was then tunneled subcutaneously and connected to a vascular access button (Instech Laboratories, Plymouth Meeting, PA). The button was exteriorized through a 1-cm incision between the scapulae. During the 7-day recovery period, the rats received daily infusions of the antibiotic Gentamycin (4 mg/kg, IV, Sigma-Aldrich, St. Louis, MO). A sterile heparin solution (0.1 mL, 50 U/mL) was flushed through the catheter before and after administering the antibiotic and after nicotine self-administration. After flushing the catheter, 0.05 mL of a sterile heparin/glycerol lock solution (500 U/mL, Instech Laboratories, Plymouth Meeting, PA) was infused into the catheter. The animals received carprofen (5 mg/kg, SC) daily for 72 h after the surgery.

### 2.5 Acquisition of nicotine self-administration

Following recovery from surgery, rats were trained to acquire nicotine self-administration (0.03 mg/kg/infusion) under a fixed ratio 1 (FR1) schedule with a 10-s timeout (TO) in sound- and light-attenuated operant chambers (Med Associates, St. Albans, VT). Rats self-administered nicotine during daily 2 h sessions for 15 sessions. Nicotine self-administration sessions were conducted 5 days per week for 3 weeks. Responding on the active lever resulted in the delivery of a nicotine infusion (0.1 mL infused over a 6.5-s period). The infusion was paired with a cue light above the active lever, which remained illuminated throughout the timeout period. Responding on the inactive lever was recorded but did not have scheduled consequences. Both levers were retracted during the timeout (TO) period.

### 2.6 Go/no-go nicotine self-administration task

The Go/No-Go self-administration task was conducted as previously described by Zapata et al. using cocaine (Zapata et al., 2017). Following the acquisition of nicotine self-administration, rats were trained to self-administer nicotine under the Go/No-Go schedule. Each session lasted 2 h and consisted of six alternating 20-min intervals: three Go periods (nicotine available; 0–20 min, 40–60 min, and 80–100 min) and three No-Go periods (nicotine unavailable; 20–40 min, 60–80 min, and 100–120 min). Responses were combined across the three Go periods and across the three No-Go periods within each session because the primary outcome was responding under nicotine-available versus nicotine-unavailable conditions, rather than changes in responding across individual intervals. Sessions always began with a Go period for all rats. Nicotine availability during the Go periods was signaled by illumination of the house light. During these periods, responding on the active lever resulted in an infusion of nicotine (0.03 mg/kg/infusion; 0.1 mL infused over 6.5 s) under a fixed ratio 5 (FR5) schedule with a 10-s timeout (TO). Each infusion was paired with a cue light above the active lever, which remained illuminated throughout the timeout period. The house light was turned off during the timeout period and turned on afterward to signal continued nicotine availability. Responses on the inactive lever were recorded but had no programmed consequences. Both levers were retracted during the timeout period. During the No-Go periods, the house light remained off to indicate that nicotine was unavailable. Lever presses were recorded during this period, but neither the active nor inactive lever produced any scheduled consequences. We assessed impulsive action using this task, specifically measuring the failure to inhibit a previously reinforced drug-directed response during the No-Go period (commission errors). This task is a well-established paradigm for evaluating motor inhibition (Bari and Robbins, 2013). Although the house light functions as a discriminative stimulus signaling nicotine availability during Go periods, impulsive action in the Go/No-Go task is operationally defined as failure to withhold responding in the presence of a well-learned No-Go signal. Impulsive responding was quantified as the percentage of active lever responses emitted during No-Go periods relative to total active lever responses across both Go and No-Go periods, calculated as [ No-Go active lever responses/(Go active lever responses + No-Go active lever responses) × 100 (Zapata and Lupica, 2021). This measure indexes commission errors, reflecting impaired inhibitory control when nicotine was unavailable. Because nicotine infusions during Go periods were obtained under an FR5 schedule, overall active lever responding was higher during Go intervals. Consequently, relying on raw counts of No-Go responses may misrepresent impulsivity, as such differences can arise from variations in overall response output or reinforcement contingencies rather than true deficits in inhibitory control. Expressing commission errors as a proportion of total active-lever responses accounts for these differences in overall response output and provides a normalized measure of response inhibition. Go/No-Go training was conducted for 41 consecutive sessions. The first 20 sessions were conducted 5 days per week, while the remaining 21 sessions were conducted 7 days per week.

### 2.7 Varenicline, nicotine, and mecamylamine treatment on go/no-go nicotine self-administration task

Following the Go/No-Go training sessions, the effects of varenicline, nicotine, and mecamylamine were assessed on nicotine self-administration behavior within the Go/No-Go task. Each drug was administered 15 min prior to the Go/No-Go nicotine self-administration (0.03 mg/kg/infusion) session. Varenicline (0, 0.1, 0.3, 1, and 3 mg/kg, SC), nicotine (0, 0.1, 0.3, and 0.6 mg/kg, SC), and mecamylamine (0, 0.5, 1, and 2 mg/kg, SC) were administered using a Latin square design. There was at least a 48-h interval between successive test doses within each drug (varenicline, nicotine, or mecamylamine). Daily Go/No-Go nicotine self-administration sessions were conducted between the treatment sessions. The treatments with nicotine began 72 h after the last varenicline session, and the treatments with mecamylamine began 72 h after the last nicotine session. Go/No-Go nicotine self-administration sessions were conducted 7 days per week.

### 2.8 Saline versus nicotine self-administration in the go/no-go task

After completion of all drug treatment experiments, a separate comparison was conducted to evaluate responding for saline versus nicotine under the Go/No-Go schedule. Rats continued to undergo daily Go/No-Go nicotine self-administration and 96 hours after the final drug treatment session, rats were assigned to a within-subject crossover design. During the first test phase, half of the animals self-administered saline, while the remaining animals self-administered nicotine (0.03 mg/kg/infusion) under identical Go/No-Go task conditions. Following this initial test, animals underwent two standard Go/No-Go nicotine self-administration sessions to re-establish baseline responding before crossing over to the alternate condition. Animals that initially self-administered saline subsequently self-administered nicotine, and animals that initially self-administered nicotine subsequently self-administered saline. Go/No-Go nicotine self-administration sessions were conducted 7 days per week.

### 2.9 Catheter patency test

Catheter patency was assessed during the self-administration period by administering 0.2 mL of the ultra-short-acting barbiturate Brevital (1% methohexital sodium). A rapid loss of muscle tone was considered indicative of a patent catheter. Rats that did not exhibit this response were excluded from subsequent analyses. One male and one female rat failed the Brevital test during the Go/No-Go training period and were excluded. Additionally, two male rats did not respond to Brevital during the nicotine treatment phase and were excluded.

### 2.10 Statistics

Data were analyzed with SPSS Statistics version 31 and GraphPad Prism version 10.5. The figures were generated using GraphPad Prism version 10.5. Lever pressing, percentage of active lever responses, nicotine intake, and infusion latency data were analyzed using two-way or three-way analysis of variance (ANOVA) with repeated measures where appropriate. The factors included treatment, sex, session, and lever (active vs. inactive). Significant interactions and main effects were further examined using the Bonferroni *post hoc* test. The alpha level for statistical significance was set at p < 0.05.

## 3 Results

### 3.1 Acquisition of nicotine self-administration

Male and female rats acquired nicotine self-administration over 15 sessions. During this period, both male and female rats responded more on the active lever than the inactive lever (Supplementary Figure S1A, Lever: F (1,22) = 31.191, P < 0.001; Sex: F (1,22) = 0.033, NS; Lever × Sex: F (1,22) = 1.209, NS). Inactive lever responding was stable, while active lever responding increased over time and then stabilized in both males and females (Supplementary Figure S1A, Session: F (14,308) = 4.753, P < 0.001; Session × Sex: F (14,308) = 0.787, NS; Lever × Session: F (14,308) = 2.717, P < 0.001; Lever × Session × Sex: F (14,308) = 0.771, NS). Nicotine intake initially increased and then stabilized in males and females (Supplementary Figure S1B, Session: F (14,308) = 12.583, P < 0.001; Session × Sex: F (14,308) = 1.698, NS; Sex: F (1,22) = 0.507, NS). Furthermore, the latency to the first nicotine infusion exhibited a sex-dependent pattern: in males, the latency initially increased before decreasing and stabilizing, whereas in females, it decreased early and remained stable thereafter (Supplementary Figure S1C, Session: F (14,308) = 7.266, P < 0.001; Sex: F (1,22) = 2.571, NS; Session × Sex: F (14,308) = 1.861, P < 0.05). The *post hoc* analysis revealed that females had significantly shorter first infusion latency than males on session 2 (Supplementary Figure S1C). In addition, the *post hoc* analysis showed that the latency to the first infusion in males was significantly longer on session two and significantly shorter from session six onward, compared to session one (Supplementary Figure S1C).

### 3.2 Go/no-go training

After the acquisition phase, male and female rats were trained to self-administer nicotine in a Go/No-Go task for 41 sessions.

Go period: During the Go period, active lever responses were higher than inactive lever responses, with no effect of sex (Figure 2A, Lever, F (1,20) = 137.087, P < 0.001; Sex: F (1,20) = 0.276, NS; Lever × Sex, F (1,20) = 0.078, NS). Active lever responding increased during the initial sessions and then remained relatively stable in both males and females, while inactive lever responses were relatively stable (Figure 2A, Session, F (40,800) = 4.515, P < 0.001; Session × Sex, F (40,800) = 1.042, NS; Lever × Session, F (40,800) = 2.384, P < 0.001; Lever × Session × Sex, F (40,800) = 1.35, NS). Nicotine intake quickly increased and then stabilized in both males and females (Figure 2B, Session, F (40,800) = 5.008, P < 0.001; Session × Sex, F (40,800) = 1.197, NS; Sex, F (1,20) = 0.272, NS).

**FIGURE 2:**
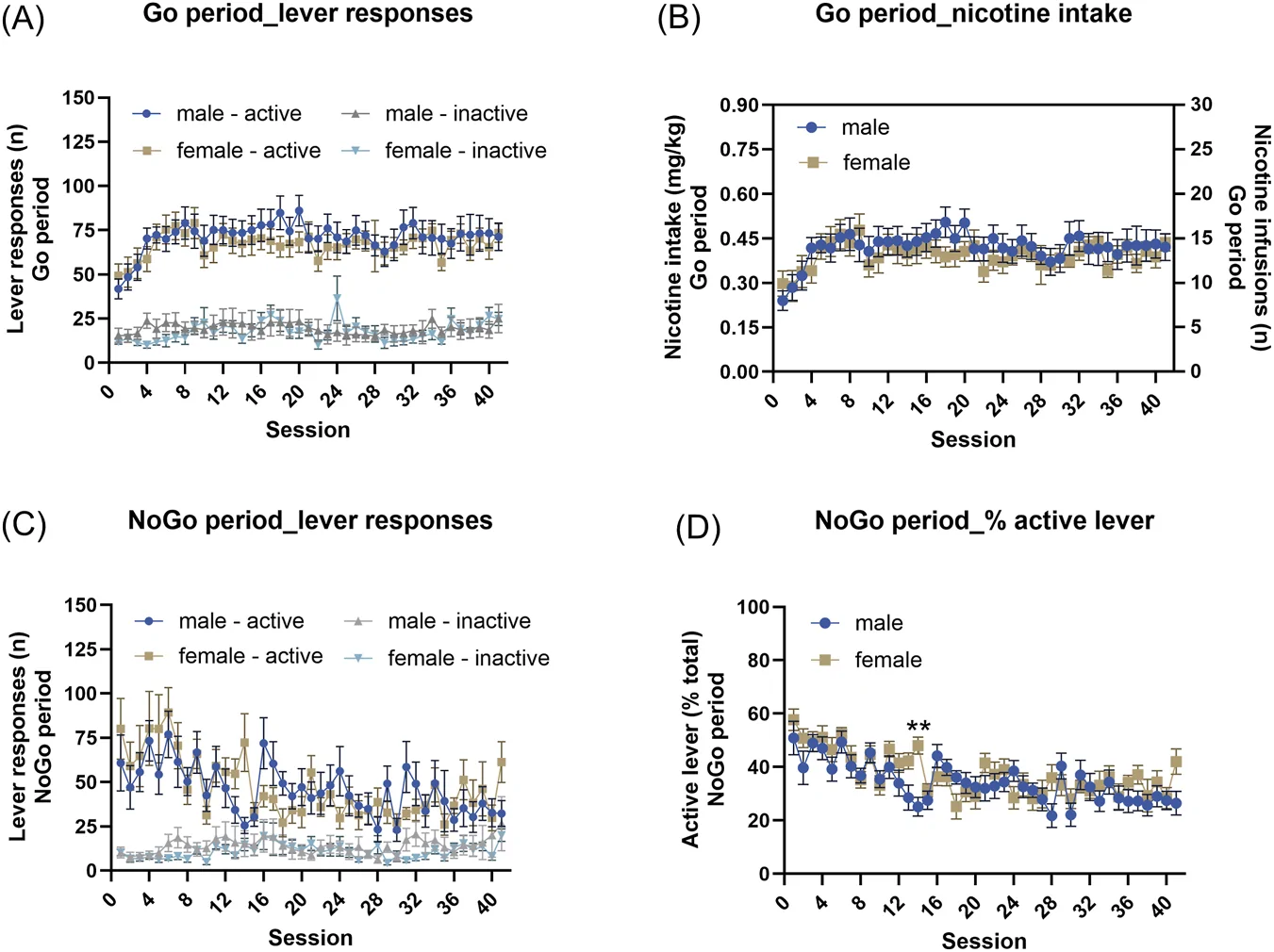
Nicotine self-administration and responding during the Go/No-Go training phase. **(A)** Active and inactive lever responses during the Go periods across the 41 training sessions. Active lever responding increased and stabilized, remaining significantly higher than inactive lever responding. **(B)** Nicotine intake (mg/kg) during the Go periods, which increased and stabilized over the training sessions. **(C)** Active and inactive lever responses during the No-Go periods. Active lever responding decreased over time, consistent with the acquisition of inhibitory control, but remained significantly higher than inactive lever responding. **(D)** Percentage of active lever responses during the No-Go periods across training sessions, which decreased over time in both sexes. Males, N = 11; Females, N = 11. **p < 0.01. Data are expressed as mean + SEM.

No-Go period: During the No-Go period, the rats responded more on the active lever than the inactive lever, with no effect of sex (Figure 2C, Lever, F (1,20) = 102.625, P < 0.001; Sex, F (1,20) = 0.024, NS; Lever × Sex, F (1,20) = 0.43, NS). Lever-pressing patterns changed significantly over time and differed between sexes (Session, F (40,800) = 4.313, P < 0.001; Session × Sex, F (40,800) = 1.745, P < 0.01; Lever × Session, F (40,800) = 5.242, P < 0.001; Lever × Session × Sex, F (40,800) = 1.768, P < 0.01). The *post hoc* analysis did not reveal significant sex differences in either active or inactive lever responses when comparing the same session between males and females. However, the *post hoc* analysis showed that the active lever responses significantly decreased from session 14 onward in males and from session 8 onward in females compared to the session one within the same sex (Supplementary Table S1). In contrast, no significant changes in inactive lever responses were observed across sessions in either sex when compared to their respective session 1.

The percentage of active lever responses during the No-Go period decreased over time and this pattern was affected by sex (Figure 2D, Session, F (40,800) = 8.144, P < 0.001; Sex, F (1,20) = 0.864, NS; Session × Sex, F (40,800) = 2.076, P < 0.01). The *post hoc* analysis showed that the females had a significantly higher percentage of active lever responses than males during session 14 (Figure 2D). In addition, the *post hoc* analysis revealed that the percentage active lever responses significantly decreased from session 12 onward in males and from session 8 onward in females compared to the session one within the same sex (Supplementary Table S1).

### 3.3 Go/no-go task: varenicline treatment

Go period: Varenicline treatment decreased both active and inactive lever responses during the Go period, and these effects were not affected by sex (Figure 3A, Active lever: Varenicline treatment, F (4,80) = 48.664, P < 0.001; Varenicline treatment × Sex, F (4,80) = 0.753, NS; Sex, F (1,20) = 0.223, NS; Figure 3C, Inactive lever, Varenicline treatment F (4,80) = 9.326, P < 0.001; Varenicline treatment × Sex, F (4,80) = 0.147, NS; Sex, F (1,20) = 0.069, NS). Furthermore, varenicline reduced nicotine intake in both males and females (Figure 3B, Varenicline treatment, F (4,80) = 46.811, P < 0.001; Varenicline treatment × Sex, F (4,80) = 0.397, NS; Sex, F (1,20) = 0.275, NS).

**FIGURE 3:**
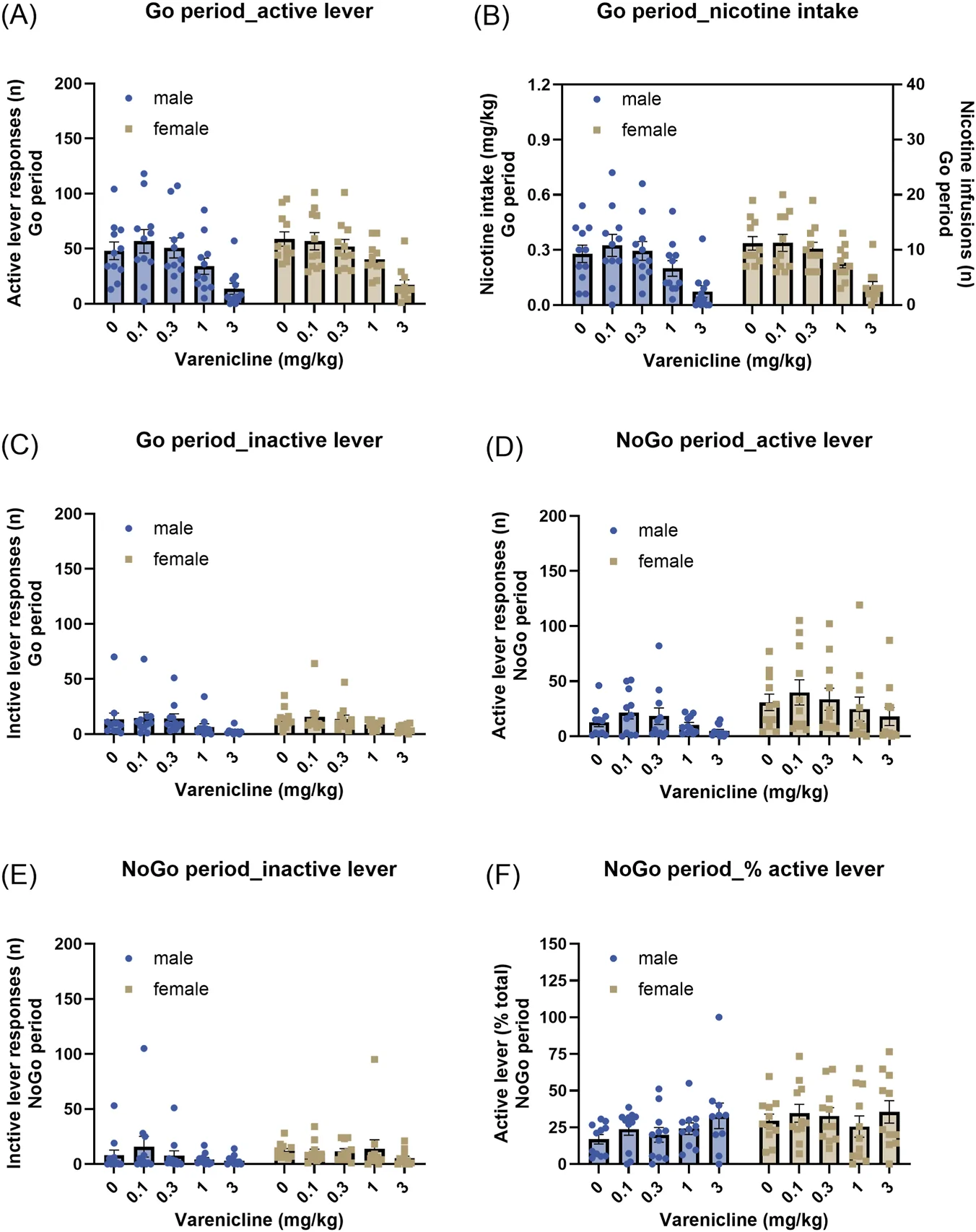
Effects of varenicline treatment on performance in the nicotine Go/No-Go task. Effects of varenicline (0, 0.1, 0.3, 1, and 3 mg/kg) on **(A)** active lever responses, **(B)** nicotine intake, and **(C)** inactive lever responses during the Go periods. Varenicline dose-dependently reduced responding and intake during Go periods. **(D)** Active lever responses, **(E)** inactive lever responses, and **(F)** the percentage of active lever responses during the No-Go periods. Varenicline decreased active lever responses during No-Go periods but did not alter the percentage of active responses (response allocation), indicating a nonspecific reduction in responding rather than specific improvement in inhibitory control. Males, N = 11; Females, N = 11. Data are expressed as mean + SEM.

No-Go period: Varenicline treatment decreased active lever responses during the No-Go period in both male and female rats (Figure 3D, Varenicline treatment, F (4,80) = 5.086, P < 0.01; Varenicline treatment × Sex, F (4,80) = 0.112, NS; Sex, F (1,20) = 3.007, NS). Inactive lever responses were not significantly affected by varenicline treatment (Figure 3E, Varenicline treatment, F (4,80) = 1.631, NS; Varenicline treatment × Sex, F (4,80) = 1.089, NS; Sex, F (1,20) = 0.327, NS). Similarly, varenicline treatment did not affect the percentage of active lever responses during the No-Go period (Figure 3F, Varenicline treatment, F (4,80) = 1.806, NS; Varenicline treatment × Sex, F (4,80) = 0.762, NS; Sex, F (1,20) = 1.975, NS).

### 3.4 Go/no-go task: nicotine treatment

Go period: Pretreatment with nicotine decreased active and inactive lever responses during the Go period, with no effects of sex (Figure 4A, Active lever: Nicotine treatment, F (3,54) = 41.204, P < 0.001; Nicotine treatment × Sex, F (3,54) = 0.501, NS; Sex, F (1,18) = 0.404, NS; Figure 4C, Inactive lever: Nicotine treatment, F (3,54) = 5.322, P < 0.01; Nicotine treatment × Sex, F (3,54) = 0.035, NS; Sex, F (1,18) = 0.04, NS). In addition, nicotine intake was decreased after nicotine treatment in both males and females (Figure 4B, Nicotine treatment, F (3,54) = 41.944, P < 0.001; Nicotine treatment × Sex, F (3,54) = 0.69, NS; Sex, F (1,18) = 0.371, NS).

**FIGURE 4:**
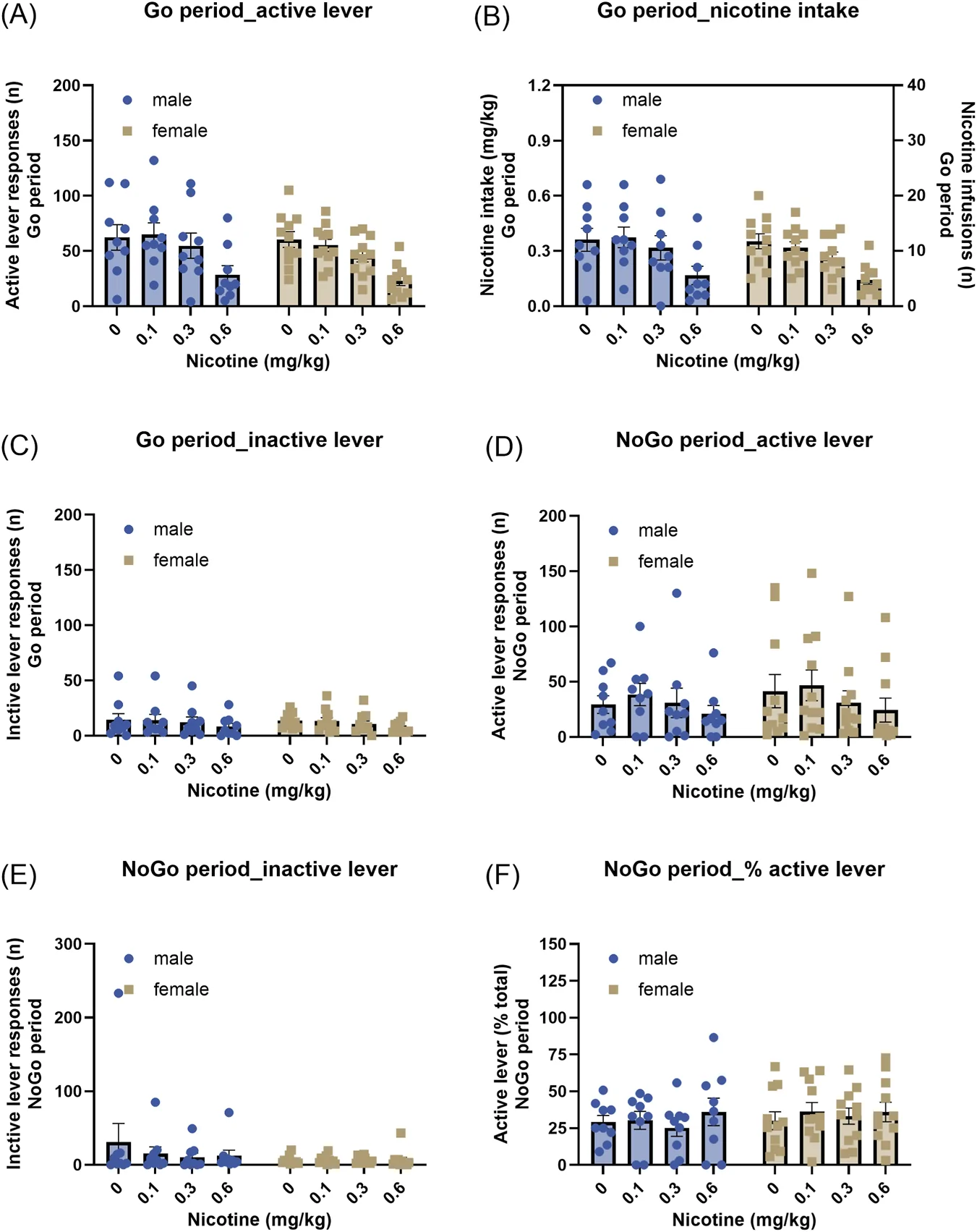
Effects of nicotine pretreatment on performance in the nicotine Go/No-Go task. Effects of nicotine pretreatment (0, 0.1, 0.3, and 0.6 mg/kg) on **(A)** active lever responses, **(B)** nicotine intake, and **(C)** inactive lever responses during the Go periods. Nicotine pretreatment decreased active responding and intake during Go periods. **(D)** Active lever responses, **(E)** inactive lever responses, and **(F)** the percentage of active lever responses during the No-Go periods. Similar to varenicline, nicotine decreased active lever responses during No-Go periods but did not alter the percentage of active responses, suggesting nonspecific suppression of behavior rather than enhanced response inhibition. Males, N = 9; Females, N = 11. Data are expressed as mean + SEM.

No-Go period: During the No-Go period, nicotine treatment decreased active lever responding in both males and females (Figure 4D, Nicotine treatment, F (3,54) = 3.471, P < 0.05; Nicotine treatment × Sex, F (3,54) = 0.351, NS; Sex, F (1,18) = 0.161, NS), while inactive lever responses were not affected (Figure 4E, Nicotine treatment, F (3,54) = 1.081, NS; Nicotine treatment × Sex, F (3,54) = 1.223, NS; Sex, F (1,18) = 1.056, NS). Furthermore, the percentage of active lever responses was not affected by nicotine treatment in either males or females (Figure 4F, Nicotine treatment, F (3,54) = 0.971, NS; Nicotine treatment × Sex, F (3,54) = 0.352, NS; Sex, F (1,18) = 0.286, NS).

### 3.5 Go/no-go task: mecamylamine treatment

Go period: Mecamylamine treatment did not affect active lever responses during the Go period, and no sex differences were observed (Figure 5A, Mecamylamine treatment, F (3,54) = 2.08, NS; Mecamylamine treatment × Sex, F (3,54) = 0.793, NS; Sex, F (1,18) = 1.512, NS). However, inactive lever responses were reduced by mecamylamine treatment, independent of sex (Figure 5C, Mecamylamine treatment, F (3,54) = 5.233, P < 0.01; Mecamylamine treatment × Sex, F (3,54) = 1.182, NS; Sex, F (1,18) = 1.429, NS). Mecamylamine produced a trend toward reduced nicotine intake, however, this effect did not reach statistical significance (Figure 5B, Mecamylamine treatment, F (3,54) = 2.749, P = 0.052; Mecamylamine treatment × Sex, F (3,54) = 0.644, NS; Sex, F (1,18) = 1.86, NS).

**FIGURE 5:**
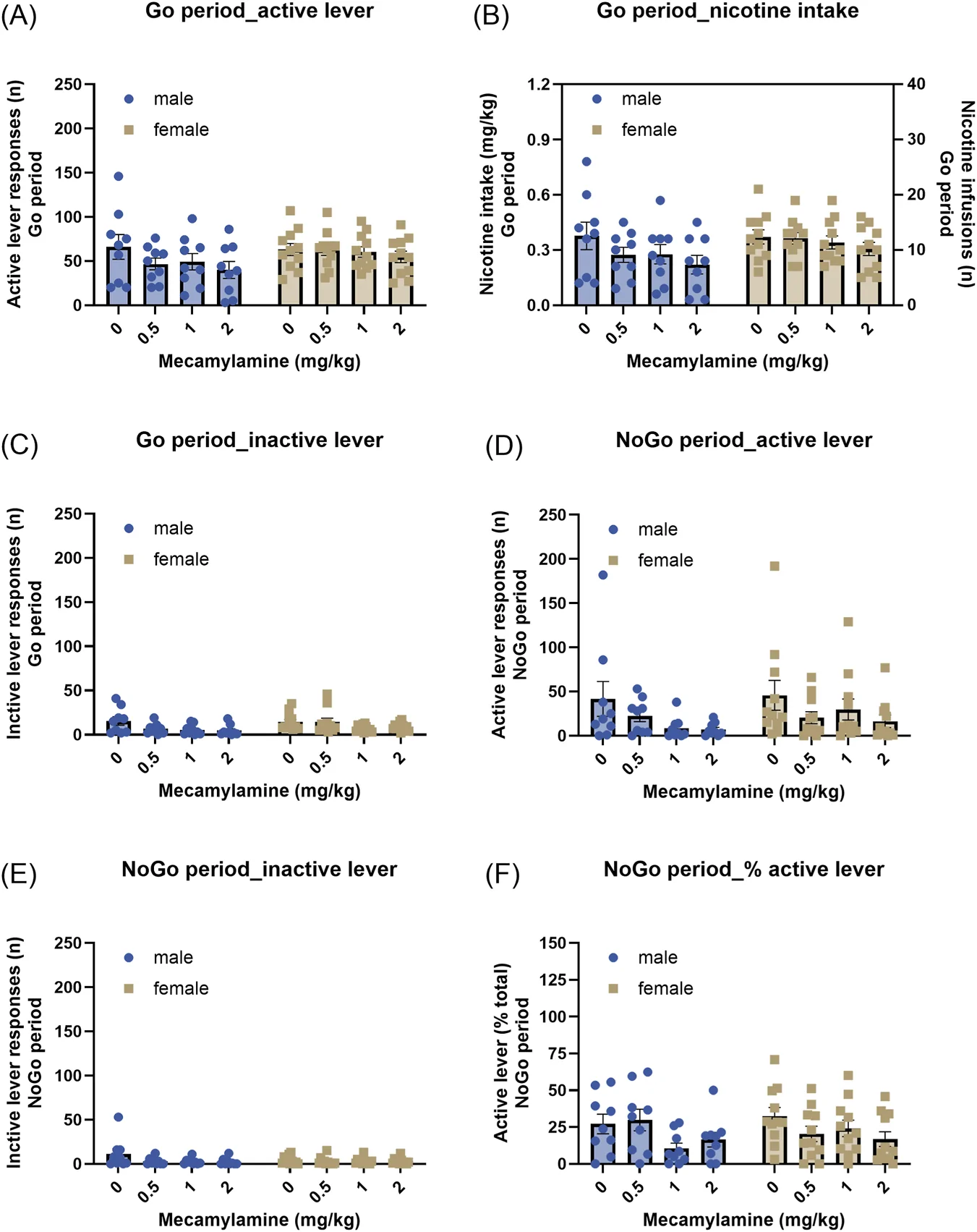
Effects of mecamylamine treatment on performance in the nicotine Go/No-Go task. Effects of mecamylamine (0, 0.5, 1, and 2 mg/kg) on **(A)** active lever responses, **(B)** nicotine intake, and **(C)** inactive lever responses during the Go periods. Mecamylamine did not significantly alter active lever responses and produced a trend toward reduced nicotine intake during Go periods. **(D)** Active lever responses, **(E)** inactive lever responses, and **(F)** the percentage of active lever responses during the No-Go periods. Mecamylamine significantly reduced both active lever responding and the percentage of active responses during No-Go periods, consistent with reduced impulsive action during nicotine self-administration. Males, N = 9; Females, N = 11. Data are expressed as mean + SEM.

No-Go period: During the No-Go period, mecamylamine treatment decreased both active and inactive lever responses in both males and females (Figure 5D, Active lever: Mecamylamine treatment, F (3,54) = 5.028, P < 0.01; Mecamylamine treatment × Sex, F (3,54) = 0.63, NS; Sex, F (1,18) = 0.483, NS; Figure 5E, Inactive lever: Mecamylamine treatment, F (3,54) = 3.532, P < 0.05; Mecamylamine treatment × Sex, F (3,54) = 2.103, NS; Sex, F (1,18) = 0.477, NS). Mecamylamine administration also decreased the percentage of active lever responses during the No-Go period (Figure 5F, Mecamylamine treatment, F (3,54) = 4.114, P < 0.05; Mecamylamine Treatment × Sex, F (3,54) = 2.35, NS; Sex, F (1,18) = 0.177, NS).

### 3.6 Go/no-go task: saline and nicotine self-administration

Go period: During the Go period, active lever responses and infusions were similar between the Nicotine Group and Saline Group, with no effects of sex (Figure 6A, Active lever: Group, F (1,18) = 0.166, NS; Group × Sex, F (1,18) = 0.547, NS; Sex, F (1,18) = 0.15, NS; Figure 6B, Infusions: Group, F (1,18) = 0.058, NS; Group × Sex, F (1,18) = 0.675, NS; Sex, F (1,18) = 0.215, NS). However, inactive lever responses during the Go period were significantly higher in the Nicotine Group compared to the Saline Group, regardless of sex (Figure 6C, Group, F (1,18) = 18.917, P < 0.001; Group × Sex, F (1,18) = 0.029, NS; Sex, F (1,18) = 1.12, NS).

**FIGURE 6:**
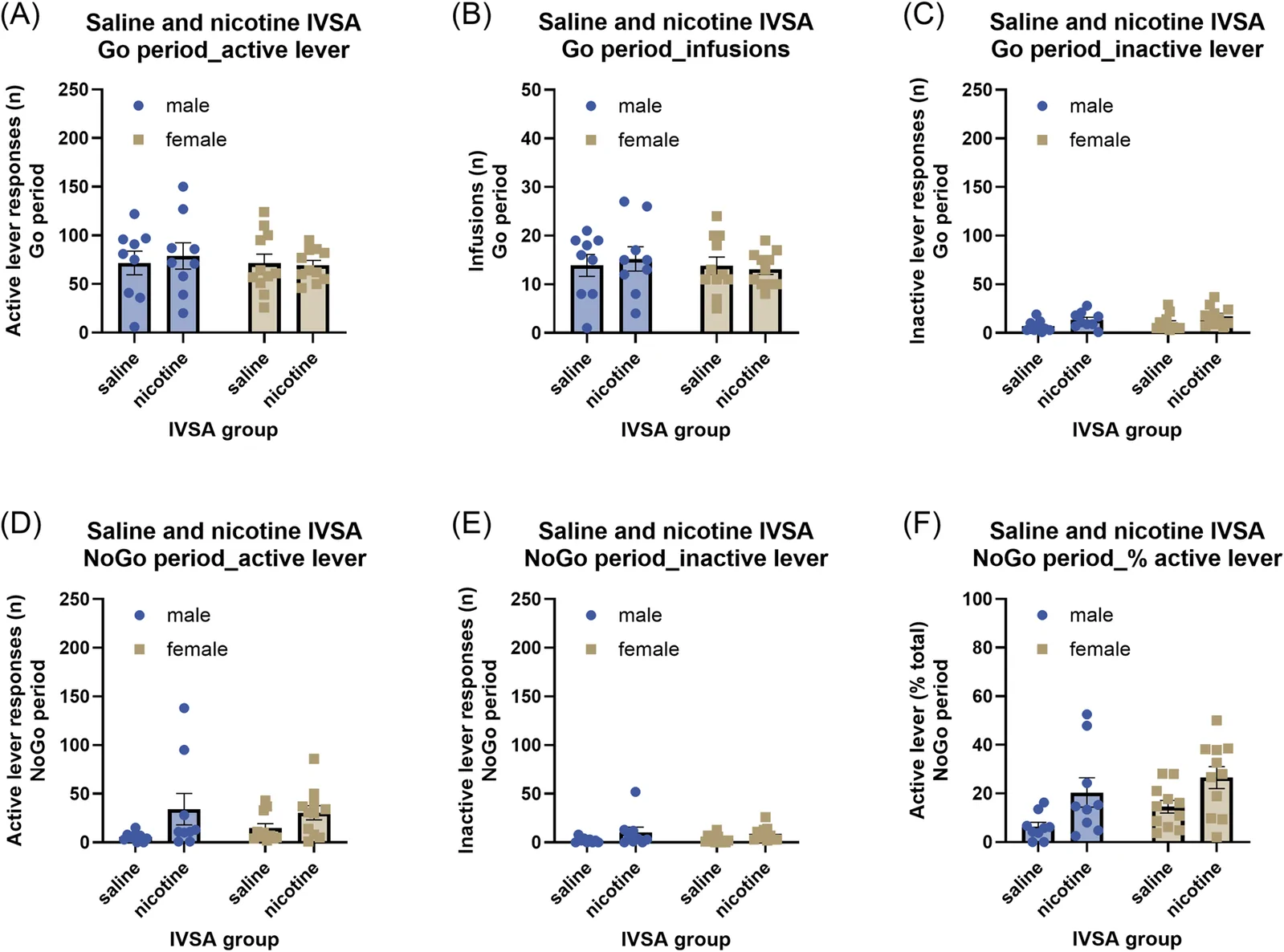
Comparison of Go/No-Go performance in rats self-administering nicotine versus saline. **(A)** Active lever responses, **(B)** total infusions, and **(C)** inactive lever responses during the Go periods for rats self-administering nicotine vs. saline. **(D)** Active lever responses, **(E)** inactive lever responses, and **(F)** percentage of active lever responses during the No-Go periods. Rats self-administering nicotine displayed significantly higher active lever responding and a higher percentage of active responses during No-Go periods compared with the saline condition. Males, N = 9; Females, N = 11. Data are expressed as mean + SEM.

No-Go period: During the No-Go period, rats in the Nicotine Group had more active and inactive lever responses than those in the Saline Group, with no significant sex differences (Figure 6D, Active lever: Group, F (1,18) = 8.022, P < 0.05; Group × Sex, F (1,18) = 0.677, NS; Sex, F (1,18) = 0.087, NS; Figure 6E, Inactive lever: Group, F (1,18) = 5.403, P < 0.05; Group × Sex, F (1,18) = 0.35, NS; Sex, F (1,18) = 0.003, NS). Similarly, the percentage of active lever responses during the No-Go period was higher in the Nicotine Group compared to the Saline Group, independent of sex (Figure 6F, Group, F (1,18) = 14.012, P < 0.001; Group × Sex, F (1,18) = 0.091, NS; Sex, F (1,18) = 2.522, NS).

## 4 Discussion

In this study we investigated whether nAChR modulators affect impulsive action in a nicotine self-administration Go/No-Go task in male and female rats. The present study demonstrates that nAChR modulators differentially affect impulsive action during nicotine self-administration. During the Go/No-Go training period, responses on the active lever increased during Go periods and decreased during No-Go periods, indicating effective acquisition of both the ‘go’ response and inhibitory control. The rats also displayed higher active than inactive lever responses during No-Go periods, indicating high levels of impulsive action under conditions requiring inhibitory control. Acute pretreatment with varenicline or nicotine reduced active lever responding during both Go and No-Go periods, but did not alter response allocation, indicating that these reductions reflect nonspecific decreases in responding rather than improvements in inhibitory control. The non-selective nAChR antagonist mecamylamine did not affect active lever responses during the Go period but decreased active lever responding during No-Go periods. Finally, substituting saline for nicotine infusions decreased active lever responding during No-Go periods without affecting active lever responding during Go periods. These findings emphasize the utility of the Go/No-Go paradigm for investigating nAChR-dependent modulation of impulsive action during nicotine self-administration.

In the present study, we found that nicotine self-administration increased responding during the No-Go period and increased the percentage of active responses during the No-Go period compared with the saline self-administration. However, saline does not provide the reinforcing effects of nicotine, and therefore this comparison does not isolate a specific effect of nicotine on impulsive action. This observation is in line with a previous Go/No-Go study that investigated the effects of noncontingently administered nicotine on impulsive behavior (Kolokotroni et al., 2011). Kolokotroni et al., investigated the effects of nicotine in a symmetrically reinforced Go/No-Go task and a systematic delayed reward task (Kolokotroni et al., 2011). Acute nicotine administration induced a dose-dependent increase in both impulsive choice and increased responding during No-Go trials indicating increased impulsive action. Studies using the 5-CSRTT, a well-established assay for assessing visuospatial attention and impulsive action, also indicate that nicotine treatment affects impulsive behavior (Asinof and Paine, 2014). For example, Day et al. reported that repeated nicotine exposure decreased response latencies and increased inappropriate responding, indicative of heightened impulsivity in rats previously exposed to nicotine (Day et al., 2007). Similarly, Blondel et al. found that acute and repeated nicotine treatment increased anticipatory responding in rats (Blondel et al., 2000). While these studies establish that passively administered nicotine can impair inhibitory control, our study extends this work by assessing impulsive action within a voluntary self-administration paradigm. This design allows nicotine intake and impulsive action to be assessed within the same session, a context more relevant to human tobacco use.

In the present study we also investigated the effects of the nAChR modulators nicotine, mecamylamine, and varenicline on responding in the Go/No-Go paradigm. Pretreatment with nicotine decreased nicotine intake during the Go period. Consistent with previous findings, pretreatment with nicotine dose-dependently reduced nicotine self-administration, likely due to nAChR occupancy or desensitization that diminishes the reinforcing effects of nicotine (Corrigall and Coen, 1989; Green et al., 2000; Dani, 2015). Pretreatment with nicotine also led to a decrease in active lever responses during the No-Go period; however, because the percentage of active responses was unchanged, this decrease likely reflects reduced overall responding rather than enhanced inhibitory control. Previous studies indicate that nicotine-induced impulsive action is primarily mediated by activation of nAChRs (Blondel et al., 2000; Kolokotroni et al., 2011). In the present paradigm, both nicotine pretreatment and self-administered nicotine likely engaged the same nAChR-dependent mechanisms during task performance, resulting in sustained receptor activation despite reduced nicotine intake. As a result, nicotine pretreatment did not change nicotine-associated impulsive action, consistent with the maintenance of impaired inhibitory control driven by ongoing nAChR activation. These findings indicate that reductions in nicotine reinforcement can occur independently of improvements in inhibitory control and highlight the importance of nAChR signaling in nicotine-associated impulsive action in the Go/No-Go self-administration task.

In the present study, we also investigated the effects of the nAChR antagonist mecamylamine on nicotine intake during the Go period and impulsive responding during the No-Go period. Mecamylamine did not significantly affect active lever presses during the Go period, and while there was a trend toward reduced nicotine intake, this effect did not reach statistical significance. Conflicting findings have been reported regarding the effects of mecamylamine on responding for nicotine (Corrigall and Coen, 1989; Chellian et al., 2024a; b). Mecamylamine has been shown to decrease nicotine in rats with a relatively short history of nicotine intake (Corrigall and Coen, 1989). In contrast, mecamylamine increases nicotine intake in nicotine dependent rats with a long history of nicotine intake (Chellian et al., 2024b). In our study, treatment with mecamylamine also decreased responding during the No-Go period; combined with the observed reduction in the percentage of active responses, this indicates that mecamylamine reduced impulsive action in rats that self-administered nicotine. This finding is consistent with previous reports that nicotine-induced impulsivity is attenuated by mecamylamine pretreatment (Blondel et al., 2000; Kolokotroni et al., 2011). These effects likely reflect nAChR blockade within pathways involved in impulsive action during nicotine self-administration and support a role for nAChR signaling in this behavior. Although we did not test mecamylamine in saline self-administering rats in this study, prior work suggests that mecamylamine does not alter impulsive behavior in drug-naive animals tested in symmetrically reinforced Go/No-Go tasks, systematic delayed reward tasks, or the 5-CSRTT (Blondel et al., 2000; Kolokotroni et al., 2011). Together, these findings support a role for nAChR signaling in impulsive action during nicotine self-administration and demonstrate the utility of this model for investigating pharmacological modulation of impulsive action.

We also investigated the effects of varenicline on responding during the Go and No-Go period. Varenicline (a partial agonist at α4β2 nAChRs and a full agonist at α7 receptors) is an FDA approved smoking cessation drug and decreases nicotine withdrawal and intake in rats (Faessel et al., 2010; Igari et al., 2014). In our study, varenicline decreased active lever responses during the Go period and during the No-Go period. The observation that varenicline decreases responding during the Go period is in line with previous studies showing that varenicline decreases nicotine self-administration (O'Connor et al., 2010; George et al., 2011). This study also showed that varenicline decreases responding during the No-Go period; however, the lack of change in the percentage of active responses indicates that varenicline did not modify impulsive action but instead reduced responding nonspecifically. These results are consistent with prior work using the 3-choice serial reaction time tasks (3-CSRTT), which showed that varenicline pretreatment does not alter nicotine-induced impulsive behavior (Ohmura et al., 2017). Although varenicline was not tested in saline self-administration, previous studies suggest that it can induce impulsive responding in drug-naïve animals in both 3- and 5-CSRTT paradigms, an effect attributed to its partial agonism at α4β2 nAChRs (Wouda et al., 2011; Ohmura et al., 2017). Partial agonists bind to the same receptor sites as full agonists such as nicotine but produce a submaximal receptor response; this partial activation of nAChRs has been shown to increase impulsive action in models of inhibitory control. In our paradigm, varenicline’s acute partial activation of nAChRs likely reduced nicotine-maintained responding without affecting inhibitory control, consistent with the unchanged percentage of active responses during No-Go periods. Because varenicline competes with nicotine for nAChR binding yet only partially activates these receptors, the receptor signaling necessary for impulsive action remains engaged even as nicotine intake decreases. Together, these findings indicate that reductions in nicotine intake reflect decreased reinforcement rather than modulation of impulsive action during nicotine self-administration and further support the validity of the Go/No-Go self-administration model for investigating nAChR-mediated impulsive action.

The percentage of active responses during No-Go trials served as a normalized measure of failures to withhold active-lever responding when nicotine was unavailable. Neither nicotine nor varenicline pretreatment altered this percentage, indicating these treatments did not modify the relative allocation of active-lever responding between Go and No-Go periods. In other words, they did not alter the proportion of active-lever responding emitted during No-Go periods. Thus, the unchanged percentage indicates that these reductions were not accompanied by improved response inhibition. By contrast, mecamylamine significantly reduced the percentage of active responses, consistent with reduced impulsive action during nicotine self-administration. Although mecamylamine also reduced some measures of overall responding, the reduction in this percentage indicates fewer failures of response inhibition relative to total active-lever responding. Collectively, these findings demonstrate that only mecamylamine reduced this measure of impulsive action under No-Go conditions. Although we did not observe consistent sex differences, estrous cycle stage was not monitored in females. Therefore, potential effects of hormonal fluctuations on impulsive action cannot be excluded.

In conclusion, this study demonstrates differential effects of nAChR modulators on impulsive action in a nicotine self-administration Go/No-Go task. Our findings show that drugs targeting nAChRs affect performance in the Go/No-Go task. However, only mecamylamine reduced impulsive action as measured by the percentage of active responses. Nicotine and varenicline reduced overall responding without altering response allocation. Furthermore, the nicotine and saline self-administration conditions differed in No-Go performance, although this comparison does not isolate a nicotine-specific effect because saline does not provide the reinforcing effects of nicotine. This work highlights the paradigm’s value for modeling the interaction between active drug-taking and impulsive action, providing a framework to investigate nAChR-dependent mechanisms and potential therapeutics that target this relationship in tobacco use disorder.

## Data Availability

The raw data supporting the conclusions of this article will be made available by the authors, without undue reservation.
