# Supplementary material for: Differential effects of nAChR modulators on impulsive action in a nicotine self-administration Go/No-Go task in rats
Source: Front Pharmacol. 2026 Sep 21;17:1883945. doi: 10.3389/fphar.2026.1883945 (PMC13635303; doi:10.3389/fphar.2026.1883945)
Supplement: Supplementary file 1 [file Image1.pdf]

Figure S1

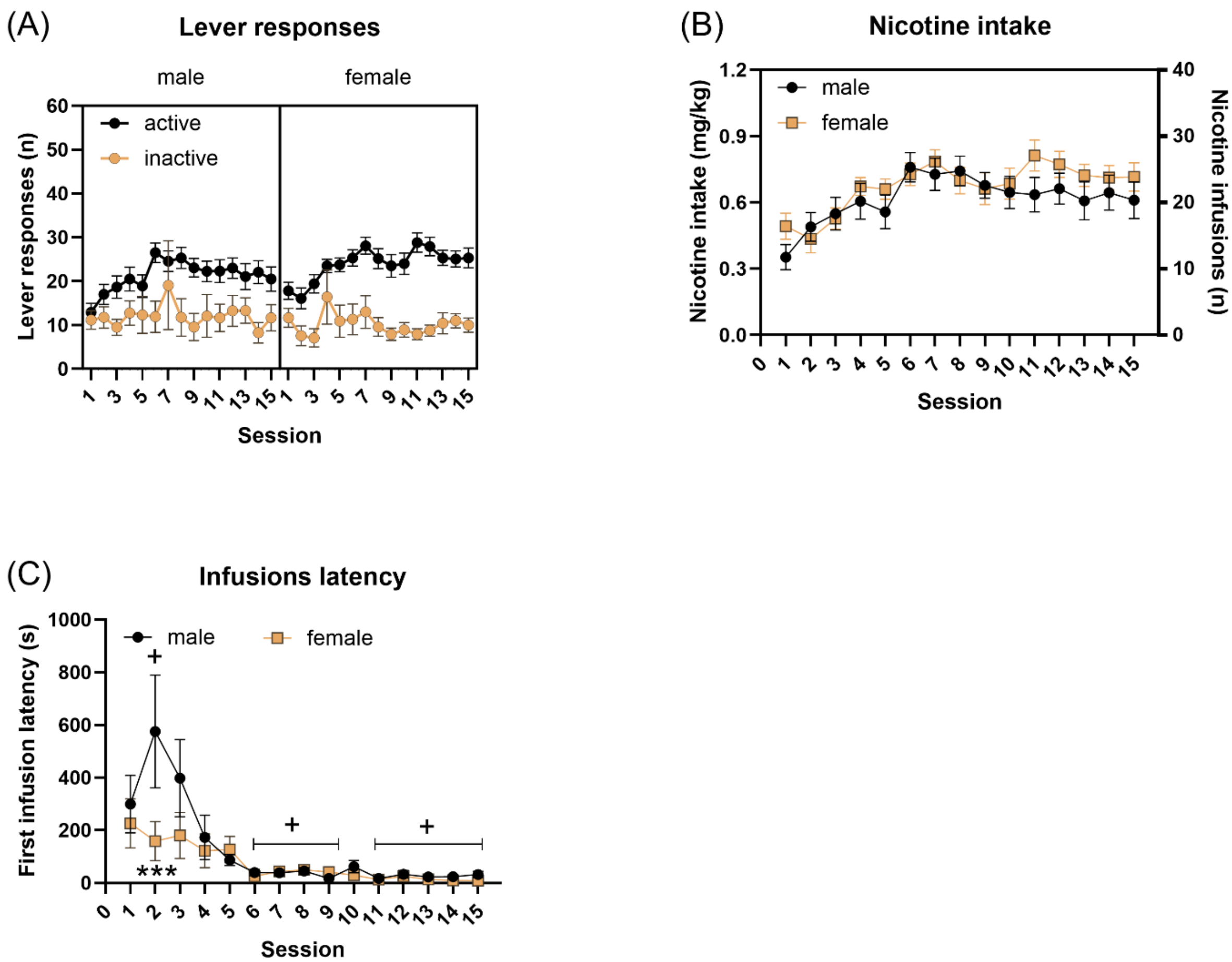

Figure S1. Spontaneous acquisition of nicotine self-administration in male and female rats. The rats self-administered nicotine (0.03 mg/kg/inf) during 2-hour self-administration sessions for 15 sessions. (A) Active lever responses, (B) inactive lever responses and (C) and latency to first infusion during nicotine self-administration sessions. Asterisks indicate shorter latency to the first infusion in females compared with the males on the same session day. Plus signs indicate significant difference in latency to the first infusion relative to first session in males. Males, N=12; Females, N=12. + P<0.05; *** P<0.001. Data are expressed as means ± SEM.
