# Supplementary material for: Differential effects of nAChR modulators on impulsive action in a nicotine self-administration Go/No-Go task in rats
Source: Front Pharmacol. 2026 Sep 21;17:1883945. doi: 10.3389/fphar.2026.1883945 (PMC13635303; doi:10.3389/fphar.2026.1883945)
Supplement: Supplementary file 2 [file Table1.docx]

**Table S1: NoGo period lever responses (compared to session 1).**

| **Session** | **Fig. 2C: NoGo period: lever responses** | | **Fig. 2D: NoGo period: percentage active lever responses** | |
| --- | --- | --- | --- | --- |
|  | **males** | **females** | **males** | **females** |
| **2** | ns | ns | ns | ns |
| **3** | ns | ns | ns | ns |
| **4** | ns | ns | ns | ns |
| **5** | ns | ns | ns | ns |
| **6** | ns | ns | ns | ns |
| **7** | ns | ns | ns | ns |
| **8** | ns | ** | ns | *** |
| **9** | ns | ns | ns | ns |
| **10** | ns | *** | ns | *** |
| **11** | ns | ns | ns | ns |
| **12** | ns | ns | * | * |
| **13** | ns | ns | *** | ns |
| **14** | ** | ns | *** | ns |
| **15** | * | *** | *** | *** |
| **16** | ns | *** | ns | *** |
| **17** | ns | *** | ns | *** |
| **18** | ns | *** | ns | *** |
| **19** | ns | *** | * | *** |
| **20** | ns | *** | ** | *** |
| **21** | ns | ns | ** | * |
| **22** | ns | *** | ** | ** |
| **23** | ns | ** | * | ** |
| **24** | ns | *** | ns | *** |
| **25** | ns | *** | ** | *** |
| **26** | ns | *** | ** | *** |
| **27** | ns | *** | *** | *** |
| **28** | ** | *** | *** | *** |
| **29** | ns | *** | ns | *** |
| **30** | ** | *** | *** | *** |
| **31** | ns | *** | ns | *** |
| **32** | ns | *** | ** | *** |
| **33** | ns | *** | *** | *** |
| **34** | ns | * | * | *** |
| **35** | ns | *** | *** | *** |
| **36** | * | *** | *** | *** |
| **37** | ns | ns | *** | ** |
| **38** | * | *** | *** | *** |
| **39** | ns | ** | *** | *** |
| **40** | ns | *** | *** | *** |
| **41** | ns | ns | *** | ns |
| Bonferroni's multiple comparisons post hoc test: Asterisks indicate a significant difference from session 1 in rats of the same sex. * P < 0.05, ** P < 0.01, *** P< 0.001. Males, N=11; Females, N=11. | | | | |
